# Case report: persistent bilateral complete sciatic artery associated with aneurysmal degeneration

**DOI:** 10.1590/1677-5449.202301512

**Published:** 2024-08-09

**Authors:** Paulo Henrique Alves Togni, André Luís Santos Vaz Leite, Marcela Prando Sampaio, Beatriz Camargo Castro, Henrique Tomaz Rodrigues, Guilherme Augusto Paro

**Affiliations:** 1 Centro Universitário Padre Albino – UNIFIPA, Catanduva, SP, Brasil.

**Keywords:** anatomical variation, aneurysm, ischemia, surgical amputation

## Abstract

Persistent sciatic artery is a rare congenital anomaly, with few cases described in the literature. This study presents a case of this embryological variation observed in a patient’s lower limb circulatory system. The anatomical description is based on a review of medical records and imaging exams. This case report describes a 63-year-old female patient admitted to the emergency department complaining of severe pain in the right lower limb, with a cold, pale extremity and ecchymosis on the dorsum of the foot. Duplex ultrasound showed no detectable flow in the anterior tibial and fibular arteries and a tardus parvus pattern in the posterior tibial artery. The patient developed loss of movement and fixed cyanosis in the right foot and was referred for urgent thromboembolectomy. However, adequate reperfusion was not seen after the procedure. Angiotomography was performed on the first postoperative day, showing bilateral persistence of the sciatic artery, with aneurysmal degeneration, partially thrombosed, and no opacification of the arterial system downstream of the aneurysm. By the third postoperative day, the patient had developed areas of dry necrosis in the limb, with no perfusion to the ankle, and underwent transfemoral amputation. Despite being a rare condition, it is of great clinical importance because of the high complication rates.

## INTRODUCTION

Persistent sciatic artery (PSA) is a rare congenital anomaly, with a prevalence close to 0.05% in the general population.^1-3^ It has an elevated predisposition to aneurysmal degeneration, which is associated with significant complications. The etiology of this process is not yet clear.^2^

This research project was approved by the Research Ethics Committee at the Centro Universitário Padre Albino (UNIFIPA), under Ethics Appraisal Submission Certificate number 70496223.7.0000.5430, and consolidated opinion number 6.130.232.

## CASE DESCRIPTION

The patient was a 63-year-old, brown-skinned female admitted to the Urgent Care Center because of sudden and intense pain in the right lower limb (RLL) with onset 1 day before and with no fever or other symptoms. She had a history of systemic arterial hypertension, major depressive disorder, and metastatic breast cancer. She was taking losartan and carbamazepine and was on palliative chemotherapy. Physical examination found the right leg and foot pale and cold and presence of ecchymosis on the dorsal foot and posterior aspect of the calf. Pedal, posterior tibial, popliteal, and femoral pulses were all absent on palpation and capillary refill time exceeded 3 seconds.

The vascular surgery team was called to assess the case. Initial workup found platelets at 68,000/mm^3^ and serum sodium at 131 mEq/L. A duplex scan of the RLL arterial system showed diffuse atheromatous plaques in the arterial bed, including in the common femoral, deep femoral, and superficial femoral arteries with a triphasic flow pattern. In the popliteal artery, a triphasic flow pattern was observed in the portions that could be assessed, up to the mid third of the leg. The tardus parvus pattern was seen in the posterior tibial artery. Detectable flow was also absent on the duplex scan in the anterior tibial and fibular arteries, which would indicate proximal obstruction. There was also thickened and hypoechogenic skin and subcutaneous cellular tissue, interspersed with anechoic layers, suggesting edema. On the basis of these findings, the diagnostic hypothesis was acute arterial occlusion.

The patient lost movement of the right foot, which had developed fixed cyanosis, and was sent for urgent thromboembolectomy of the RLL.

In surgery, an inguinotomy was performed, sectioning by planes until the region of the common femoral artery was reached, where an aneurysm of the great saphenous was found, with increased collaterals. The right common femoral artery was dissected, with a weak amplitude pulse present, and was looped with cardiac tape proximally and distally. Arteriotomy was performed and a 5F thromboembolectomy catheter was advanced distally to the superficial femoral artery (SFA), removing small quantities of thrombus even after several attempts. The catheter did not advance to the topography of the popliteal artery. After closure of the femoral artery, an incision was made over the dorsal artery of the right foot, which was dissected. Next, an attempt was made to advance a 4F thromboembolectomy catheter, which also could not be successfully advanced into the topography of the popliteal artery, although a moderate quantity of clotting was removed. The site was then closed. Finally, the areas were cleaned and hemostasis checked. At the end of the procedure, the patient’s RLL was still cold and perfusion impaired, and the limb was bandaged.

**Figure 1:**
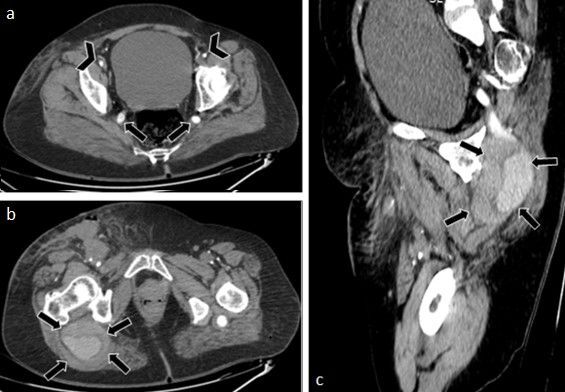
Computed tomography (CT) – abdominal window. (a) Axial. Bilateral persistence of the sciatic artery, representing continuation of the internal iliac artery to the popliteal artery (complete type) via the greater sciatic foramen, posterior to the piriformis muscle, along the path of the sciatic nerve (black arrow). There is associated bilateral hypoplasia of the external iliac arteries and the femoral system, maintaining opacification (arrowhead); (b) Axial. Aneurysmal degeneration (possibly of a chronic traumatic nature) of the right sciatic artery, originating around 7.0 cm below the internal iliac artery, 9.2 cm in length, with a maximum axial diameter of 6.7 × 5.7 cm. The aneurysmal degeneration has lobulated and irregular margins (black arrow); (c) Sagittal. Aneurysmal degeneration (possibly of a chronic traumatic nature), partially thrombosed, of the right sciatic artery. There is also an absence of opacification of the arterial system downstream from the aneurysm in the phases available (black arrow).

**Figure 2:**
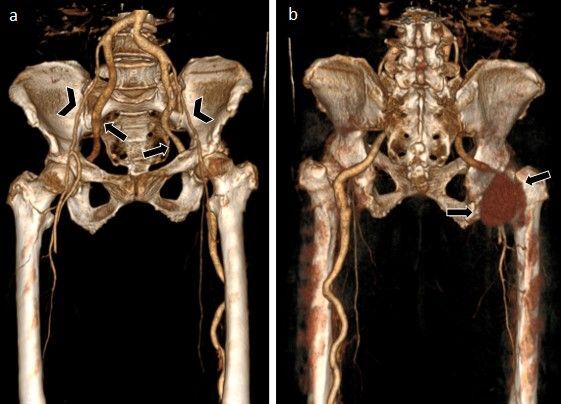
3D reconstruction. (a) Anterior. Bilateral persistence of the sciatic artery, representing continuation of the internal iliac artery to the popliteal artery (complete type) via the greater sciatic foramen, posterior to the piriformis muscle, along the path of the sciatic nerve (black arrow). There is associated bilateral hypoplasia of the external iliac arteries and the femoral system, maintaining opacification (arrowhead); (b) Posterior. Aneurysmal degeneration (possibly of a chronic traumatic nature) of the right sciatic artery originating around 7.0 cm below the internal iliac artery, 9.2 cm in length, with a maximum axial diameter of 6.7 × 5.7 cm (black arrow).

At the first postoperative physical examination, the patient stated she was not in pain, her RLL temperature was low, she had no pedal or posterior tibial pulses, the popliteal pulse was strong, and the femoral pulse was of low amplitude. Her toes were rigid, with loss of sensitivity and movement, fixed cyanosis, and long capillary refill time. The appearance of the surgical wound was good. She was kept on anticoagulation with the limb bandaged. Angiotomography (ACT) was ordered, which revealed bilateral persistent complete sciatic artery. This was a continuation of the internal iliac artery to the popliteal artery via the greater sciatic foramen, posterior to the piriformis muscle, along the path of the sciatic nerve (Figure 1). There was also bilateral hypoplasia of the external iliac arteries and the femoral system, with opacification maintained (Figure 2). Partially thrombosed aneurysmal degeneration involving the right sciatic artery was identified, arising around 7.0 cm below the internal iliac artery and 9.2 cm in length. Downstream from the aneurysm, there was no opacification of the arterial system.

On the second postoperative day, the patient’s condition was as on the previous day, although with changed toe color. A meeting with the patient’s family was held and authorization was requested to amputate the limb. Administration of heparin was suspended.

On the third postoperative day, the limb still had no pulse and there were signs of fibrosis and mumification, with no perfusion to the ankle and areas of dry necrosis. A right transfemoral amputation was necessary.

After the surgery, the appearance of the amputation stump was good. Postoperative recovery was free from intercurrent conditions and the patient was discharged 4 days after the amputation. A physiotherapy team home visit was requested to support readaptation to daily activities and the patient was instructed to rest until an outpatient appointment 14 days later.

At the outpatient reassessment, she was asymptomatic and the surgical wound was dry with no signs of necrosis or secretion in response to pressure. Alternate sutures were removed and the patient was requested to return in 20 days. At the next consultation, the remaining sutures were removed and 75 mg pregabalin was prescribed for pain in the stump, with reassessment scheduled for 6 months. After this date, the patient did not return for outpatient consultations.

The Case Report guidelines were followed when writing this report.

## DISCUSSION

Persistent sciatic artery is a rare embryological anomaly, with incidence of 0.01 to 0.06%^1-3^ of the population. A bibliographic review published by Mann et al.^1^ in 2022 compiled 102 cases of this malformation reported from 1964 to 2021.^1^ The first report of this condition was published by Green in 1832, who described it as a variant of the femoral artery.^4,5^

Embryologically, the lower limb bud emerges in the embryo at 5 mm and is supplied by the umbilical artery. At 6 mm, the proximal portion of the umbilical artery is formed by 2 roots, one ventral and one dorsal. The axial artery ascends from the dorsal portion and constitutes the main blood supply to the lower extremity of the embryo at 9 mm.^6-8^ When the ventral root of the umbilical artery involutes, the external iliac artery ascends proximal to the axial artery, with the embryo measuring 8.5 mm.

At 12 mm, the external iliac artery supplies the femoral artery and the femoral plexus. During this stage, there are two systems supplying the lower limb, a ventral, comprising the external iliac artery, and a dorsal, formed by the sciatic artery.^7^

Beyond 22 mm, the blood flow via the femoral artery increases and the sciatic artery involutes naturally. This series of events is completed around the third month of gestation.^7,9,10^

With regard to PSA, Bower et al.^11^ proposed a classification of complete or incomplete, distinguishing them by the pattern of supply to the lower limb. In the complete form, which accounts for 63 to 79% of cases, the sciatic artery is the principal source of blood supply to the lower limb, and is continuous with the internal iliac artery to the popliteal artery.^2,9,12^ The SFA is hypoplastic in the majority of cases of the complete variant.^9^ In the incomplete form, the sciatic artery is hypoplastic and terminates at the level of the internal iliac or popliteal arteries, communicating with the femoral system, which constitutes the dominant arterial supply, via collateral branches.^7,9,12^

Pillet et al.^13^ discriminated between four different types of PSA. Type 1 is a complete persistent sciatic artery in association with a normal femoral artery. Type 2 is a complete PSA in association with an incompletely developed femoral system and is subdivided into 2a and 2b. In subtype 2a, the SFA is present but does not reach the popliteal artery, whereas in type 2b, the SFA is absent. Types 3 and 4 are incomplete PSA forms, in which only the proximal or distal segment persists, respectively, and the femoral arteries develop normally. In 1994, Gauffre et al.^13^ described a type 5 PSA originating from the median sacral artery. Type 5 is also subdivided, depending on development of the SFA - the superficial femoral system is developed in type 5a and is undeveloped in type 5b.

Bibliographic reviews by Mann et al.,^1^ Yang et al.,^2^ Accarino et al.,^5^ and van Hooft et al.^13^ found that the mean age of presentation of the clinical complications of PSA is around the sixth decade of life and sex-distribution is equal. The great majority of reports, around 80%, describe complete forms of PSA.^1,2,10,12,13^ Presentation is unilateral in approximately 70 to 80%, with no preference for side. Bilateral occurrence is reported in 20 to 30%.^1,5,13^

Patients with PSA typically remain asymptomatic until aneurysm formation, which sometimes provokes complications such as thrombosis, embolization, rupture, or ischemia.^2^ Aneurysmal dilatation is present in around 40 to 61% of cases.^2^ The exact etiology is unknown, but is presumed to be multifactorial. Suggestions include chronic trauma caused by the anatomic trajectory, along which the artery is subjected to compression and friction by pelvic elements, in addition to a congenital predisposition to atherosclerosis and hypoplasia of arterial connective tissue components.^2,8,9^

The majority of patients present with symptomatic PSA (70 to 80%) and the most common clinical manifestations include: gluteal mass and/or discomfort, acute ischemia, claudication, distal embolization, compressive sciatic neuropathy, and pale and cold extremity, which may or may not all be present.^12,13^ Severe cases that are not treated in time can result in ischemic necrosis and limb loss.^1^

On physical examination, the Cowie sign is considered pathognomonic for PSA and consists of reduction or absence of the femoral pulse in combination with presence of the popliteal pulse.^2,13,14^ According to the literature, digital subtraction angiography is the imaging method most used for diagnosis of PSA, because it provides information on anatomic classification and enables complete assessment of the distal peripheral arterial system.^13,14^ In turn, ACT and magnetic resonance angiography can provide more detailed information on aneurysms, including size, anatomic relationships, and path.^14,15^ Additionally, ACT can reveal a totally occluded artery, which cannot be seen with conventional angiography.^13-15^

Treatment of PSA depends on symptomology, presence or absence of aneurysm, and classification. Surgical management is not mandatory in asymptomatic patients, but it is recommended that they be routinely followed-up with ultrasonography, because of the elevated thromboembolic risk.^2,13-15^

Management of symptomatic complete or incomplete PSA, with no aneurysm, demands revascularization, since the vessel constitutes the main blood supply to the lower limb. Patients that can undergo open surgery may benefit from femoral-popliteal bypass and for those that need a less invasive procedure, endovascular repair with PSA stenting is a viable option.^2,13,16^ According to the literature, treatment of a complete aneurysmal PSA demands both revascularization and exclusion of the aneurysm, which can be by ligature, embolectomy, thrombectomy, and/or endoaneurysmorrhaphy. The most commonly employed revascularization procedure is femoral-popliteal bypass, excluding the PSA, but interposition bypass graft and stenting are also possible. For an incomplete, symptomatic, aneurysmal PSA, management also involves exclusion of the aneurysm and bypass, if necessary, or use of stenting.^2^

Endovascular interventions have been used for revascularization, exclusion of aneurysm, and treatment of focal atherosclerotic lesions of the sciatic artery.^13,16^ A review conducted by Charisis et al.^16^ in 2020 demonstrated that patients who underwent endovascular treatment had a very low rate of post-procedural complications.

Despite the many therapeutic methods, there is not yet consensus on treatment of this rare condition.^5,12^

## CONCLUSIONS

Persistent sciatic artery is a rare congenital anomaly with severe clinical repercussions if not managed in time. There is not yet consensus on management of this disease, because of its rarity. Nevertheless, clinical presentation and vessel morphology and classification are good guides for individualization of treatment and patient follow-up. Endovascular management has proved promising for this condition and can contribute to reducing postoperative complications.
