# Supplementary material for: Case report: persistent bilateral complete sciatic artery associated with aneurysmal degeneration
Source: J Vasc Bras. 2024 Aug 9;23:e20230151. doi: 10.1590/1677-5449.202301512 (PMC11404772; doi:10.1590/1677-5449.202301512)
Supplement: Translation

# Translation: Relato de caso: persistência da artéria isquiática completa bilateral associada à degeneração aneurismática

**DOI:** 10.1590/1677-5449.202301511

**Authors:** Paulo Henrique Alves Togni, André Luís Santos Vaz Leite, Marcela Prando Sampaio, Beatriz Camargo Castro, Henrique Tomaz Rodrigues, Guilherme Augusto Paro

**Affiliations:** 1 Centro Universitário Padre Albino – UNIFIPA, Catanduva, SP, Brasil.

**Keywords:** variação anatômica, aneurisma, isquemia, amputação cirúrgica

## Abstract

A persistência da artéria isquiática é uma anomalia congênita rara, com poucos casos descritos na literatura. Este estudo tem como objetivo apresentar a variação embriológica observada no sistema circulatório de membros inferiores da paciente observada. A descrição anatômica foi realizada a partir da revisão de prontuário e exames de imagem. O presente caso relata uma paciente feminina, de 63 anos de idade, admitida em serviço de pronto atendimento com queixa de dor importante em membro inferior direito, com extremidade fria e pálida e presença de equimose em dorso do pé. Realizou-se *duplex scan* que evidenciou ausência de fluxo detectável nas artérias tibial anterior e fibular, e padrão *tardus parvus* na artéria tibial posterior. A paciente evoluiu com perda de movimentação e cianose fixa no pé direito e foi encaminhada para tromboembolectomia de urgência, entretanto, após o procedimento, não foi visualizado reperfusão adequada. No primeiro pós-operatório, foi realizada angiotomografia, que evidenciou persistência da artéria isquiática bilateralmente, com degeneração aneurismática parcialmente trombosada e ausência de opacificação do sistema arterial à jusante do aneurisma. No terceiro dia pós-operatório, a paciente apresentava áreas de necrose seca em membro, sem perfusão até tornozelo, e foi submetida à amputação transfemoral. Apesar de ser uma condição rara, apresenta grande importância clínica devido a suas altas taxas de complicações.

## INTRODUÇÃO

A persistência da artéria isquiática (PAI) é uma anomalia congênita rara, com uma prevalência próxima de 0,05% na população geral^1-3^ . A PAI tem elevada predisposição à degeneração aneurismática, que é associada a importantes complicações. A etiologia do processo ainda não é clara^2^ .

O presente trabalho foi aprovado pelo Comitê de Ética em Pesquisa do Centro Universitário Padre Albino (UNIFIPA), sob Certificado de Apresentação de Apreciação Ética número 70496223.7.0000.5430, e número do parecer consubstanciado 6.130.232.

## DESCRIÇÃO DE CASO

A paciente tinha 63 anos de idade, era do sexo feminino, parda, e foi admitida em pronto atendimento devido a dor intensa e súbita em membro inferior direito (MID) há 1 dia, sem febre ou demais sintomas. Relatava histórico de hipertensão arterial sistêmica, transtorno depressivo maior e câncer de mama metastático. Além disso, fazia uso de losartana e carbamazepina, e realizava quimioterapia paliativa. Ao exame físico, a perna e o pé direitos apresentavam-se pálidos, frios, com presença de equimose em região dorsal do pé e em face posterior de panturrilha. Os pulsos pedioso, tibial posterior, poplíteo e femoral não eram palpáveis, e o tempo de enchimento capilar era maior que 3 segundos.

Foi solicitado avaliação da equipe de cirurgia vascular. Na abordagem inicial, a avaliação laboratorial discriminou plaquetas de 68.000/mm^3^, sódio sérico de 131 mEq/L. Posteriormente, foi realizado *duplex scan* do sistema arterial do MID, que revelou placas ateromatosas difusas no leito arterial avaliado, incluindo a artéria femoral comum, femoral profunda e femoral superficial com fluxo de padrão trifásico. Na artéria poplítea, foi observado fluxo padrão trifásico nas porções passíveis de avaliação, até terço médio da perna. Padrão *tardus parvus* na artéria tibial posterior. Verificou-se, também, ausência de fluxo detectável ao *duplex scan* nas artérias tibial anterior e fibular, podendo representar obstrução à montante. Além disso, havia espessamento e hipoecogenicidade da pele e do tecido celular subcutâneo, com lâminas anecoicas de permeio, o que inferia edema. A partir dessas evidências, a hipótese diagnóstica era de oclusão arterial aguda.

Paciente evoluiu com perda de movimentação e cianose fixa no pé direito, sendo submetida a tromboembolectomia de urgência de MID.

No ato cirúrgico, foi realizado inguinotomia e secção por planos até topografia de artéria femoral comum, onde houve achado de aneurisma de safena magna com aumento de colaterais. A artéria femoral comum direita foi dissecada, com presença de pulso de pequena amplitude, e laçada com fita cardíaca proximal e distalmente. Foi realizada arteriotomia e passagem de cateter de tromboembolectomia 5F distalmente para artéria femoral superficial (AFS), com saída de pequeno trombo mesmo após várias tentativas. O cateter não progrediu em topografia de artéria poplítea. Após o fechamento de artéria femoral, realizou-se incisão em topografia de artéria dorsal do pé direito e dissecação da mesma. Em seguida, foi realizada tentativa de passagem de cateter de tromboembolectomia 4F, também sem sucesso na progressão em topografia de artéria poplítea, porém com saída de uma quantidade moderada de coágulos. Posteriormente, foi realizado o fechamento local. Por fim, foi realizada lavagem breve e revisão de hemostasia. Ao final do procedimento, a paciente ainda apresentava MID frio e com perfusão prejudicada, sendo então realizado o enfaixamento do membro.

Ao exame físico do primeiro pós-operatório (PO), a paciente negou queixas álgicas, apresentava MID com diminuição da temperatura, ausência de pulsos pedioso e tibial posterior, presença de amplo pulso poplíteo e pulso femoral com baixa amplitude. Também foi observada rigidez de pododáctilos, com perda da sensibilidade e movimentação, cianose fixa e tempo de enchimento capilar alargado. A ferida operatória apresentava bom aspecto. Foi mantida conduta de anticoagulação e enfaixamento de membro. Solicitou-se uma angiotomografia (ATC), na qual foi visualizada persistência da artéria isquiática completa bilateralmente. Isso representa a continuação da artéria ilíaca interna com a artéria poplítea através do forame ciático maior, posterior ao músculo piriforme, ao longo do trajeto do nervo ciático (Figura 1). Em adição, havia hipoplasia de artérias ilíacas externas e do sistema femoral bilateral, mantendo opacificação preservada (Figura 2). Na artéria isquiática direita, foi identificada degeneração aneurismática, parcialmente trombosada, a qual se originava cerca de 7,0 cm abaixo da artéria ilíaca interna, com extensão de 9,2 cm. À jusante do aneurisma, não havia opacificação do sistema arterial.

**Figura 1:** Tomografia computadorizada (TC) – janela de abdome. (a) Axial. Persistência da artéria isquiática bilateralmente, representando continuação da artéria ilíaca interna com a artéria poplítea (tipo completo) através do forame ciático maior, posterior ao músculo piriforme, ao longo do trajeto do nervo ciático (seta preta). Associa-se hipoplasia das artérias ilíacas externas e do sistema femoral bilateralmente, mantendo opacificação preservada (cabeça de seta); (b) Axial. Degeneração aneurismática (de possível natureza traumática crônica) da artéria isquiática direita, originando-se cerca de 7,0 cm abaixo da artéria ilíaca interna, com extensão de 9,2 cm, apresentando diâmetro axial máximo de 6,7 × 5,7 cm. A degeneração aneurismática apresenta contornos lobulados e irregulares (seta preta); (c) Sagital. Degeneração aneurismática (de possível natureza traumática crônica), parcialmente trombosado, da artéria isquiática direita. Nota-se ainda ausência de opacificação do sistema arterial à jusante do aneurisma nas fases disponíveis (seta preta).

**Figura 2:** Reconstrução 3D. (a) Anterior. Persistência da artéria isquiática bilateralmente, representando continuação da artéria ilíaca interna com a artéria poplítea (tipo completo) através do forame ciático maior, posterior ao músculo piriforme, ao longo do trajeto do nervo ciático (seta preta). Associa-se hipoplasia das artérias ilíacas externas e do sistema femoral bilateralmente, mantendo opacificação preservada (cabeça de seta); (b) Posterior. Degeneração aneurismática (de possível natureza traumática crônica) da artéria isquiática direita, originando-se cerca de 7,0 cm abaixo da artéria ilíaca interna, com extensão de 9,2 cm, apresentando diâmetro axial máximo de 6,7 × 5,7 cm (seta preta).

Ao segundo PO, as condições do dia anterior foram mantidas, entretanto, agora com alteração da coloração de pododáctilos. Foi realizada uma conversa com a família da paciente e solicitado autorização para a amputação do membro. A administração de heparina foi suspensa.

No terceiro PO, o membro seguia sem apresentar pulso, com sinais de fibrose e mumificação sem perfusão até o tornozelo, apresentando áreas de necrose seca, com necessidade de submeter paciente à amputação transfemoral direita.

Após realização cirúrgica, o coto de amputação evoluiu com bom aspecto. O PO ocorreu sem intercorrências, e quatro dias após amputação, a paciente recebeu alta. Foi solicitado avaliação domiciliar para readaptação das atividades diárias com equipe de fisioterapia, e orientação de repouso relativo até retorno ambulatorial em 14 dias.

Na reavaliação ambulatorial, a paciente estava assintomática, com ferida operatória seca e sem sinais de necrose ou saída de secreção à expressão. Realizou-se retirada de pontos intercalados e foi solicitado retorno da paciente em 20 dias. Durante nova consulta, os pontos restantes foram retirados e foi prescrito pregabalina 75 mg devido a queixa de dor em membro residual, com reavaliação marcada para 6 meses. Após essa data, a paciente não retornou mais ao ambulatório.

As diretrizes da Case Report foram seguidas para a elaboração deste relato.

## DISCUSSÃO

A PAI é uma anomalia embriológica rara, com incidência de 0,01 a 0,06%^1-3^ da população. A revisão bibliográfica realizada por Mann et al.^1^ , em 2022, compilou 102 casos reportados dessa malformação, datados de 1964 a 2021^1^ . A primeira descrição dessa condição foi feita por Green, em 1832, como uma variante da artéria femoral^4,5^ .

Embriologicamente, o broto do membro inferior surge no embrião de 5 mm, e seu suprimento deriva da artéria umbilical. Aos 6 mm, a porção proximal da artéria umbilical é formada por 2 raízes, uma ventral e outra dorsal. Da porção dorsal, ascende a artéria axial, que representa o principal suprimento sanguíneo da extremidade inferior no embrião de 9 mm^6-8^ . Quando a raiz ventral da artéria umbilical involui, ascende, próximo a artéria axial, a artéria ilíaca externa, com o embrião medindo 8,5 mm.

Com 12 mm, a artéria ilíaca externa supre a artéria femoral e o plexo femoral. Durante esse estágio, existem dois sistemas irrigando o membro inferior, um ventral, composto pela artéria ilíaca externa, e outro dorsal, formado pela artéria isquiática^7^ .

Após os 22 mm, o fluxo sanguíneo pela artéria femoral aumenta e, naturalmente, a artéria isquiática involui. Por volta do terceiro mês de gestação, essa série de eventos é concluída^7,9,10^ .

Em relação à PAI, Bower et al.^11^ propuseram sua classificação em completa ou incompleta, distinguindo-as quanto ao padrão de irrigação para o membro inferior. Na forma completa, que ocorre em 63 a 79% dos casos, a artéria isquiática é a principal fonte de irrigação para o membro inferior, estando em continuidade com a artéria ilíaca interna até a artéria poplítea^2,9,12^ . Na maioria dos casos da variante completa, a AFS encontra-se hipoplásica^9^ . Já na forma incompleta, a artéria isquiática encontra-se hipoplásica e é interrompida no nível da ilíaca interna ou poplítea, comunicando-se com o sistema femoral, que constitui o suprimento arterial dominante, por meio de ramos colaterais^7,9,12^ .

Pillet et al.^13^ discriminaram quatro tipos diferentes de PAI. O tipo 1 representa a persistência da artéria isquiática na sua forma completa em associação com a artéria femoral normal. O tipo 2 refere-se à PAI completa em associação com um desenvolvimento incompleto do sistema femoral, subdividido em 2ª e 2b. No subtipo 2ª, a AFS está presente, mas não alcança a artéria poplítea, enquanto no 2b, a AFS está ausente. Os tipos 3 e 4 consistem na PAI incompleta, onde apenas o segmento proximal ou distal persiste, respectivamente, e as femorais apresentam um desenvolvimento normal. No tipo 5, descrito por Gauffre et al.^13^ em 1994, a PAI origina-se da artéria sacral mediana. Além disso, também é subdividido, obedecendo ao desenvolvimento da AFS, onde o tipo 5ª o sistema femoral superficial encontra-se desenvolvido, enquanto no 5b, não.

De acordo com as revisões bibliográficas realizadas por Mann et al.^1^ , Yang et al.^2^ , Accarino et al.^5^ , van Hooft et al.^13^ , a idade média de apresentação das complicações clínicas da PAI está por volta da sexta década de vida, e está igualmente distribuída entre os sexos. A grande maioria dos relatos, cerca de 80%, descrevem a PAI em sua forma completa^1,2,10,12,13^ . Sua apresentação unilateral ocorre em aproximadamente 70 a 80%, sem predileção quanto ao lado. Relatos de sua ocorrência bilateral ocorrem em 20 a 30%^1,5,13^ .

Pacientes com PAI tipicamente permanecem assintomáticos até a formação do aneurisma, que, por vezes, leva a complicações como trombose, embolização, ruptura ou isquemia^2^ . A dilatação aneurismática está presente em cerca de 40 a 61% dos casos^2^ . Sua etiologia exata ainda é desconhecida, mas presume-se ser multifatorial. São sugeridas ocorrências de trauma crônico, resultante de seu trajeto anatômico, onde a artéria está sujeita à compressão e atrito contra elementos pélvicos, além da predisposição à aterosclerose e hipoplasia congênita de componentes do tecido conjuntivo da artéria^2,8,9^ .

A maioria dos pacientes que apresentam PAI são sintomáticos (70 a 80%), e as manifestações clínicas mais comuns incluem: massa e/ou desconforto glúteo, isquemia aguda, claudicação, embolização distal, neuropatia isquiática compressiva e extremidade pálida e fria, sendo todos esses associados ou não^12,13^ . Casos severos, se não tratados a tempo, podem resultar em necrose isquêmica e perda do membro^1^ .

Ao exame físico, o sinal de Cowie é considerado patognomônico para a PAI e consiste na diminuição ou ausência do pulso femoral em associação com a presença do pulso poplíteo^2,13,14^ . De acordo com a literatura, em relação aos métodos de imagem, a angiografia por subtração digital é a técnica mais utilizada no diagnóstico da PAI, pois fornece informações sobre a classificação anatômica e avaliação completa sobre o sistema arterial periférico distal^13,14^ . A ATC e a angiografia por ressonância magnética podem fornecer informações mais detalhadas do aneurisma, incluindo tamanho, relações anatômicas e trajeto^14,15^ . Em adição, a ATC pode evidenciar uma artéria totalmente ocluída, padrão que não pode ser visualizado pela angiografia convencional^13-15^ .

O tratamento da PAI depende da sintomatologia, presença ou não de aneurisma, e sua classificação. Em pacientes assintomáticos, o manejo cirúrgico não é mandatório, contudo, sugere-se acompanhamento, com ultrassonografia de rotina, devido ao alto risco tromboembólico^2,13-15^ .

O manejo da PAI completa ou incompleta, sintomática, na ausência de aneurisma, requer revascularização, pois esse vaso representa o principal suprimento sanguíneo do membro inferior. Pacientes que podem ser submetidos à cirurgia aberta podem se beneficiar com um *by-pass* fêmoro-poplíteo, e para aqueles que necessitam de um procedimento menos invasivo, o reparo endovascular com endoprótese na PAI é uma opção viável^2,13,16^ . Pela literatura, o tratamento da PAI completa aneurismática necessita tanto de revascularização quanto de exclusão do aneurisma, que inclui os métodos de ligadura, embolectomia, trombectomia e endoaneurismorrafia. O procedimento de revascularização mais comumente empregado foi a cirurgia de *by-pass* fêmoro-poplíteo excluindo o trajeto da PAI, entretanto, enxerto de interposição em ponte e endoprótese também são opções possíveis. Na PAI incompleta aneurismática, na presença de sintomatologia, o manejo também inclui exclusão do aneurisma e realização de *by-pass,* se necessário, ou utilização de endoprótese^2^ .

Intervenções endovasculares têm sido usadas para revascularização, exclusão aneurismática e o tratamento de lesões ateroscleróticas focais da artéria isquiática^13,16^ . A revisão realizada em 2020 por Charisis et al.^16^ demonstrou que pacientes submetidos ao tratamento endovascular apresentaram um índice de complicação pós procedimento muito baixo.

Apesar das várias modalidades terapêuticas, ainda não há consenso quanto ao tratamento dessa rara condição^5,12^ .

## CONCLUSÃO

A PAI é uma anomalia congênita rara com repercussões clínicas graves se não manejada habilmente. Dada a raridade dessa doença, ainda não existe consenso sobre seu manejo. Apesar disso, a apresentação clínica, a morfologia do vaso e sua classificação são bons guias na individualização do tratamento e acompanhamento do paciente. O manejo endovascular tem se mostrado promissor na correção desta condição, além de contribuir para a diminuição das complicações pós-operatórias.
